# *Schistosoma mansoni* infection and its association with nutrition and health outcomes: a household survey in school-aged children living in Kasansa, Democratic Republic of the Congo

**DOI:** 10.11604/pamj.2018.31.197.16364

**Published:** 2018-11-21

**Authors:** Madeleine Mbuyi Kabongo, Sylvie Linsuke, Sylvain Baloji, Faustin Mukunda, Inocêncio da Luz Raquel, Christine Stauber, Jean-Pierre Van Geertruyden, Pascal Lutumba

**Affiliations:** 1 Georgia State University, Atlanta, USA; 2 National Institute of Biomedical Research (INRB), Department of Epidemiology, Kinshasa, Democratic Republic of Congo; 3 Programme National de Lutte Contre la Trypanosomiase Humaine Africaine, Kinshasa, Democratic Republic of Congo; 4 Programme National de Lutte Contre la Bilharziose et Parasitoses Intestinales, Kinshasa, Democratic Republic of Congo; 5 Global Health Institute, University of Antwerp, Belgium; 6 Tropical Medicine Department, University of Kinshasa, Kinshasa, Democratic Republic of Congo

**Keywords:** Schistosomiasis, risk factors, nutritional status, epidemiology, Democratic Republic of Congo

## Abstract

**Introduction:**

Schistosomiasis (SCH) is an important public health problem in developing countries and school-aged children are the most affected. This study explored health and nutritional status and their correlation with SCH in children attending primary school (3rd to 6th class) living in the area of Kasansa in the Democratic Republic of Congo.

**Methods:**

Across-sectional household survey was carried out in Kasansa health area in February 2011. Children whose parents reported to attend primary school (3^rd^ to 6^th^ class) were included. Socio-demographic characteristics, information on morbidity history and risk factor were collected using a semi-structured questionnaire. *S. mansoni* and malaria infection were assessed using the Kato-katz technique and rapid diagnostic test, respectively. Haemoglobin concentration was also performed using a portable HemoControl device. Bivariate and multiple logistic regressions were used to assess risk factors for *S. mansoni*.

**Results:**

A total of 197 school aged children participated in the study with a median age of 12 years and 53.8% of them were boys. The overall health status of the children was poor with very high prevalences of *S. mansoni* infection (89.3%), malaria infection (65.1%), anaemia (61.4%) and stunting (61.0%). Regular contact with river water was the most important risk factor (OR: 11.7; p<0.001) related to SCH infection. A low haemoglobin concentration was significantly associated with a SCH infection (OR: 12.3; p=0.003) and egg load was associated with stunting (OR: 12.4; p=0.04). Children from farmers were more at risk for low school performance (OR: 5.3; p=0.03).

**Conclusion:**

High prevalence of *Schistosoma mansoni* and malaria infection was observed in the study population living in Kasansa area. Moreover, they presented a high burden of anaemia, chronic malnutrition and low school performance. An integrated disease control and management of these diseases and their consequences, endorsed by surveillance, is needed.

## Introduction

Schistosomiasis (SCH) remains a serious public health problem in developing countries with a humid tropical climate [1, 2]. It is listed as one of the neglected tropical diseases (NTD) and is poverty related [3, 4]. It is the second most prevalent tropical disease, after malaria, causing severe morbidity in large areas of the world [5]. Approximately 800 million people may be at risk of infection worldwide and more than 200 million are infected, leading to the loss of up to 4.4 million disability-adjusted life years (DALYs), of which 90% are in sub-Saharan Africa [6, 7]. Poor communities without access to safe water and adequate sanitation are more infected since agricultural, domestic and recreational activities expose them to infested water [8, 9]. When in contact with fresh water, the cercaria, the larva of the schistosome, penetrate the skin; enter the capillaries and lymphatic vessels to migrate further to the mesenteric system or vesical plexus where they develop to adult worms in the blood vessels [10]. The morbidity of SCH is mainly caused by detrimental inflammatory reactions to the eggs trapped in the tissues of the gastrointestinal or genitourinary tract [2]. Most persons infected with schistosomes do not suffer from severe hepatosplenic disease (caused by *S. mansoni* and *S. japonicum*) or bladder calcification and hydronephrosis (caused by *S. haematobium*), but from less dramatic morbidities. Therefore, the overall impact on DALYs lost due to the lower grade pathologies is higher than those lost due to severe, sometimes life threatening pathologies [11].

Of all age groups, school aged children (aged between 6 and 15 years) are the most exposed because they are traditionally responsible for water-related household chores and because they often spend their free time swimming [12]. This age group also suffers the most. Chronic infection has alarming effects on their growth and also contributes to chronic anaemia, diminished school performances and even cognitive disorders [11]. Children with heavy worm burden and poor nutritional status are most likely to suffer from cognitive impairment [13]. Moreover, chronic SCH infection is commonly associated with anaemia [14]. A recent study conducted in the health wone of Kasansa showed that school aged children living in this area are highly infected by SCH [15]. In some areas, the prevalence of SCH infection was up to 94%. This high burden may have severe implications on the children's health status but unfortunately this study did not assess these. The aim of the present study is filling this gap and assessed the SCH related morbidity in the hyper-endemic health area that was reported with a prevalence of >90%. Some risk factors associated to SCH infection and associations with other health outcomes such as malnutrition, anaemia and low school performance were also assessed.

## Methods

### Ethical considerations

Ethical clearance was obtained from the ethical committees of the World Health Organization (WHO) (reference project ID A61119) and of the school of public health, Kinshasa, Democratic Republic of Congo (reference number: ESP/CE/025/2007). District health and education authorities, the chief of the village and parents or legal guardians of the children were informed about the purpose, procedures, potential risks and benefits of the study. The school-aged children were also informed. Written informed consent was obtained from the parents or legal guardians before inclusion of the children. Children who tested positive for *S. mansoni* were treated with a single dose of praziquantel (40mg/Kg body weight), according to the WHO guidelines. Children with a positive malaria result and/or presenting aneamia were referred to the health center of Kasansa for treatment according to the national guidelines.

### Study area and population

**Figure 1:**
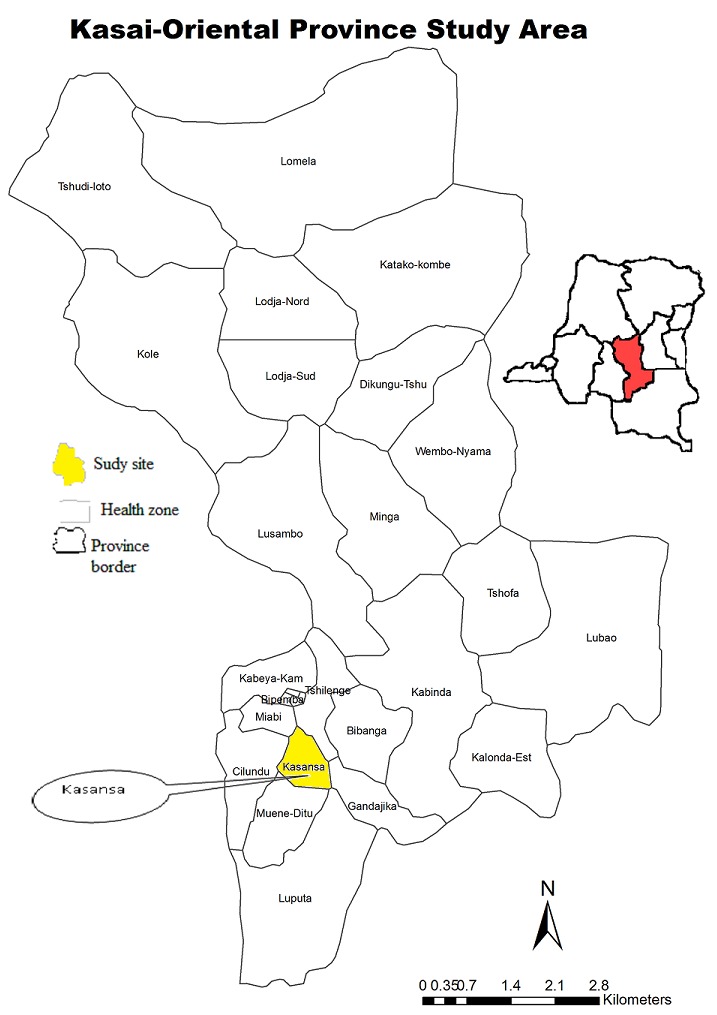
Study area, the province of Kasaï Oriental, Democratic Republic of the Congo

This study was held in February 2011 in the health zone of Kasansa, located in the Kasaï Oriental province in the Democratic Republic of Congo (DRC) (Figure 1). This health zone has an estimated 191,986 inhabitants and is located in the South-West of Kasaï Oriental province (Figure 1). The population mainly lives from agriculture, domestic small livestock, fishing, small business and artisanal exploitation of diamond. The inhabitants depend on streams and rivers for their sources of water for domestic purposes and several rivers cross the region including the Lufingala, Nsenga-senga, Muya, Mulunguyi, Monzo, Mbanda and Lac Lomba. The health area Kasansa was randomly chosen among the 4 health areas of the Kasansa health zone which presented a prevalence of >90% in the study of Linsuke *et al*. 2014 [15].

### Study design

This was a cross-sectional, household survey in which children attending primary school (3^rd^ to 6^th^ class) were targeted. At the time of the study, all children were at home since it was holidays. Therefore the household survey was organized and the children were only included when they were present in the household and reported to attend primary school. The investigators went to the central point of Kasansa and a spinning bottle was used to randomly choose the direction to follow for door to door visits. The sample size was based on the WHO guidelines on SCH surveys [16] and 203 children that reported to attend primary school (3^rd^ to 6^th^ class) were included during a household survey. Age, sex, weight and height were recorded for each participating child. Information on morbidity history (previous abdominal pain, diarrhea, blood in the stool and previous treatment of SCH), risk factors (social economic status, behaviour, sanitary conditions, exposure habits) and school attendance, were also obtained from the parents or caretakers by standard questionnaires. A stool sample was collected from each participating child to determine the presence and intensity of SCH and the other intestinal helminths. A finger prick blood sample was taken to determine haemoglobin concentration and malaria infection.

### Laboratory analysis

#### S. mansoni infection

Kato-Katz technique [17] was performed for the detection of SCH infection. One stool sample was provided and slides of Kato-Katz were made in two fold and read by two different technicians 24 hours after their preparation. Infection load was calculated as the average number of eggs found between the slides. The intensity of infection was defined by eggs per gram of faeces according to the WHO classification [16]: light infection: 1-99 eggs/gram of faeces, moderate infection: 100-399 eggs/gram of faeces, heavy infection: ≥400 eggs/gram of faeces

### Haemoglobin concentration and anaemia

Haemoglobin (Hb) concentration was determined using a portable photometer (HemoCue^®^ Hb 201) according to the manufacturer's instruction. The WHO thresholds were used for classification [18, 19]: children were anaemic if Hb concentration was <11.5g/dl for children 7-11 years of age, <12.0g/dl for 12-14 years of age, <12.0g/dl and <13.0g/dl for respectively females and males older than 15 years.

### Malaria infection

Malaria infections were detected using the commercial rapid diagnostic test (RDT) SD Bioline^®^ Malaria Ag Pf/Pan (Standard diagnostics^®^, Korea) according to manufacturer's instructions.

### Data analysis

Data were double-entered and validated in EPI INFO version 3.5.1 software and analysed using STATA version 12.0 (STATA Corp, Lakeway, College Station, Texas, USA). Categorical variables were expressed as proportions while quantitative variables were presented as the mean ± standard deviation (SD) or median ± interquartile range (IQR) if the data were not normally distributed. WHO AnthroPlus software was used to calculate anthropometric indices' z-scores and determine the nutritional status of the school-aged children. Age in months, height in cm and weight in kg were used to calculate the following indicators: 1) height-for-age Z score (HAZ) to assess stunting; 2) weight-for-age Z-score (WAS) to assess underweight in children <10 years old and 3) body-mass-index-for-age Z-score (BAZ) to assess thinness. According to the 2007 WHO growth reference for school-aged children and adolescents, stunting, underweight and thinness were defined as <-2 SD HAZ, WAZ and BAZ respectively. Severe stunting, underweight and thinness were defined as <-3 SD HAZ, WAZ and BAZ respectively.

The analytical approach examined associations between SCH and several outcome variables: anaemia, malnutrition and low school performance Contingency tables were produced for categorical variables and Pearson's X² test was used to compare two proportions. The odds ratios (OR) and their 95% confidence intervals (CI 95%) were calculated in bi-variate analysis and p-values <0.05 were considered as significant. Multivariate logistic regression was subsequently performed with forward inclusion and backwards selection of variables to determine adjusted odds ratio (AOR) for the associations with a binary outcome variable (anaemia, malnutrition, school performance). For the continuous outcome variable Hb-concentration, One-way ANOVA was used to analyze the association with the variables included in the model that can influence Hb-concentration. The means ± SD were calculated and p-values <0.05 were considered to be significant. Linear regression model was applied in the multivariate analysis.

## Results

### General characteristics of the study population

During the study, 203 children attending 3^th^ to 6^th^ primary school class were included in the sample; 6 were excluded because of lack of stool samples and 1 for being an adult (21 years old). Out of the 197 remaining, 175 (88.8%) were living in the Kasansa village and 22 (11.2%) were living in the Lac Lomba village. Socio-demographic characteristics are presented in Table 1. The gender distribution was 53.8% males and 46.3% females. The median age was 12 years old (IQR: 11-14), with a minimum of 7 and a maximum of 17 years old.

**Table 1:** Characteristics of children attending primary school in the health zone of Kasansa, DR Congo

*N of participants* (197)	n (%)
**Demographics**	
Male	106 (53.8)
Mean age ± SD	12.1 ± 2.2
Age groups (years)	
7-9y	23 (11.7)
10-12y	96 (48.7)
≥13y	78 (39.6)
**Socio-economic status: profession chief household**	
Agriculture	153 (78.5)
Commercant	10 (5.1)
civil servant	30 (15.4)
Pastor	2 (1.0)
Missing	2 (1.0)
Mean number of persons per household ± SD	9.6 ± 0.3
Median daily expenses per person USD (IQR)	0.55 (0.45–0.69)
**water contact**	
*water to drink*	
Well	34 (17.3)
Source	156 (79.2)
Unknown	7 (3.6)
*Water to bathe*	
Well	30 (15.2)
Source	33 (16.8)
River	127 (64.5)
Unkown	7 (3.6)
***Water to wash clothes and dishes***	
Well	29 (14.7)
Source	22 (11.2)
River	138 (73.0)
Unknown	8 (4.1)
**Sanitation**	
*Presence toilette/latrine*	183 (92.9)
*Plumbing*	0 (0.0)
**stool samples**	
Prevalence S. mansoniinfection	**176 (89.3)**
Intensity of S. mansoni infection	
Light	23 (11.7)
Moderate	44 (22.3)
High	**109 (55.3)**
**Blood samples**	
Prevalence malaria infection (n=175)	**114 (65.1)**
Prevalence anaemia	**121 (61.4)**
Median Hb concentration + IQR	11.3 ± 1.8

None of the households had water taps while latrines were present in 92.9% of the households. Drinking water was derived from the nearest source (79.2%) or well (17.3%). The primary source of water for bathing was the nearest river (64.5%), source (16.8%) or well (15.2%). To wash clothes and dishes, the nearest river (70.1%) was again reported as the primary water source, followed by the nearest well (14.7%) or source (11.2%). The main profession of the chief of the household was agriculture (78.5%) followed by civil servants (15.4%) and the median number of persons per household was 9 (IQR: 7-11). The median daily expense per person in a household was 0.55 USD (IQR: 0.45-0.69). Overall, 91.8% of the children lived in a household with daily expenses per person less than 1 USD.

### Parasitic infection

### Prevalence and intensity of *S. mansoni infection*

The prevalence of SCH infection was 89.3% (CI 95%: 84.9-93.6), egg density was high in 55.3% of the children, 22.3% had moderate and 11.7% had a light egg density in the stool. In the 3 months prior to the visit, 73.6% of the children reported to suffer from abdominal pain, 50.2% had diarrhea and 41.6% had blood in the stool. Additional analysis of these symptoms associated with *S. mansoni* was performed; however, none of these parameters were found to be significantly associated with SCH infection.

### Prevalence of malaria infection

The prevalence of malaria infection was 65.1% (CI 95%:58.1-72.3) in the study population although 54.3% reported to have slept under an Insecticide-treated Bed Net (ITN). Nearly all households started using ITN after 2010 (99.0%) and the majority indicated that they received the ITN from the HZ of Kasansa (85.9%) in 2011 during the national campaign. Other sources of ITN were the health center (14.8%), and a minority had bought them from the local market (5.6%).

### Anaemia and nutritional status of school-aged children

**Figure 2:**
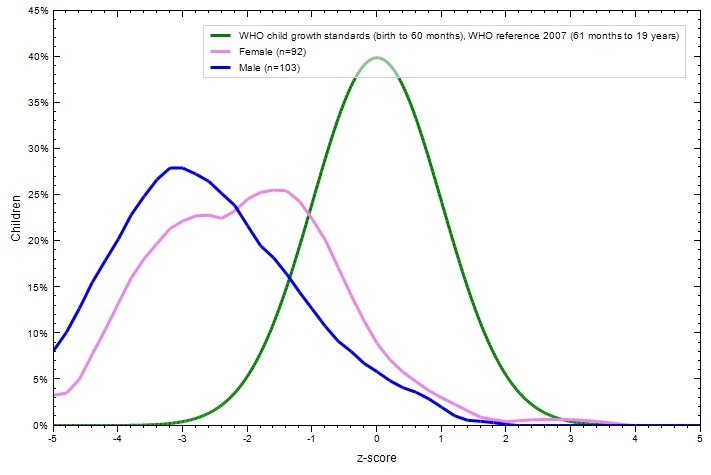
HAZ score in children attending primary school (3^rd^ to 6^th^ class) in the health zone of Kasansa in DR Congo (n=197)

The proportion of children that presented anaemia was 61.4% with a median haemoglobin concentration of 11.3g/dl (IQR: 10.5-12.3). The weight parameter (WAZ) of the nutritional status can only be calculated in children younger than 10 years old (n=23) was within healthy parameters, with 3 cases being underweight (13.0%) and 3 cases being severely underweight (13.0%) (Table 2). The thinness parameter (BAZ) was also within healthy parameters for the majority of the children but 29.8% were too thin. The proportion of children with stunting was 61.0% of which 35.9% presented severe stunting. Boys were more affected by stunting than girls (Figure 2).

**Table 2:** Nutritional status of children attending primary school (3^rd^ to 6^th^ class) in the health zone of Kasansa, DR Congo

WAZ* (n=23) underweight	n (%)
Normal	17 (73.9)
Underweight	3 (13.0)
Severe underweight	3 (13.0)
**HAZ (n= 195) stunting**	
Normal	76 (39.0)
Stunting	49 (25.1)
Severe stuntig	70 (35.9)
**BAZ (n=195) thinness**	
Normal	137 (70.3)
Too thin	34 (17.4)
Severe thiness	24 (12.3)

* WAZ score can only be calculated for children younger than 10 years old

### School performance status

Class distribution of the children was as followed: 60 children (3.0%) in third grade, 80 (40.6%) in fourth grade, 59 (29.9%) in fifth grade and 52 (26.4%) in the sixth grade. The proportion of children that reported to have failed at least one class was 54.6% and the main reasons reported for failing was failing the exams (41.2%) and illness (20.6%). The inability of the parents to pay for the tuition fee also contributed in school failure for 23.7% of the cases.

### Risk factors related to *S. mansoni infection* and the related burden as anaemia, stunting or low school performance

The first model assessed risk factors related to *S. mansoni* infection. Gender, age, presence of latrine, the profession of the household chief and water contact were included in the analysis (Table 3). Only contact with water from the nearest river and water from the source were associated with SCH infection. Water contact to the source and well were predictors for SCH infection both for bathing (OR=4.2; p=0.03 & OR=6.1; p=0.03) and washing clothes and dishes (OR=11.7; p<0.001 & OR=9.9; p<0.001). Significant associations to relate risk factors to S. mansoni infection were not found using multivariate analysis.

**Table 3:** Correlates with SCH infection of children attending primary school (3^rd^ to 6^th^ class) in the health zone of Kasansa, DR Congo

	*Correlates with S. mansoni infection*				
Variables	N	n (%)	OR	95% CI	p value
**Gender**					
Male	91	97 (92.4)	2.0	0.8-5.1	0.1
Female	105	78 (85.7)	1.0		
**Age groups**					
7-9y	23	23 (100)	1.7	0.7-4.5	0.2
10-12y	96	87 (90.6)	1.0		
>=13y	77	65 (84.4)	1.0		
**Latrine at home**					
no	14	12 (85.7)	1.0		
yes	182	163 (89.5)	1.4	0.3-6.9	0.6
**Water contact to bathe**					
well	30	19 (63.3)	1.0		
source	33	29 (87.9)	4.2	1.2-15.1	**0.03**
river	126	120 (95.2)	11.7	3.8-35.3	**<0.001**
**Water contact to wash clothes and dishes**					
well	29	18 (62.1)	1.0		
source	22	20 (90.9)	6.1	1.2-31.4	**0.03**
river	137	129 (94.2)	9.9	3.5-27.8	**<0.001**
**Profession of chief houshold**					
commercant	10	8 (80.0)	1.0		
pastor	2	1 (50)	0.25	0.01-6.0	0.4
civil servant	29	28 (96.5)	7.0	0.6-87.5	0.1
agriculture	153	136 (88.9)	2.0	0.4-10.2	0.4

The second model assessed correlates of anaemia (Table 4). The variables associated with a decrease of Hb concentration were gender (p=0.02), SCH infection (p=0.003), intensity of SCH egg load (p<0.001), self-reported presence of blood in the stool (p=0.001) and stunting (p<0.001). When correlation between anaemic status (binary outcomes) and SCH infection was analyzed, self-reported blood in the stool (OR=2.2; p=0.0.01), and severe stunting (OR=2.8; p=0.004) were retained as associated (Table 4). These associations were maintained when multivariate analysis was performed. The AOR were 2.3 (p=0.008) and 1.7 (p=0.007) respectively for self-reported blood in the stool and severe stunting.

**Table 4:** Correlates with the decrease of Hb-concentration and anaemia in children children attending primary school (3^rd^ to 6^th^class) in the health zone of Kasansa, DR Congo

Variables	*Correlates with Hb-concentration*		*Correlates with anaemia*				*Predictors for anaemia*		
	Mean Hb (SD)	p value	n (%)	OR	95% CI	p value	AOR	95% CI	p value
**Gender**									
Male	11.6 (5.6)		67 (63.8)	1.3	0.7-2.3	0.4			
Female	11.4 (6.2)	**0.02**	53 (58.2)	1					
**Agegr**									
7-9y	11.2 (5.6)	0.4	15 (65.2)	1.0	0.4-1.3	0.3			
10-12y	11.4 (5.8)		55 (57.3)	0.7	0.4-2.7	0.9			
>=13y	11.6 (6.1)		50 (64.9)	1					
**SCH infection**									
no	12.3 (6.2)	**0.003**	10 (47.6)	1					
yes	11.4 (5.7)		110 (91.7)	1.8	0.4-2.1	0.2			
**SCH density**									
0	12.4 (6.3)	**<0.001**	10 (47.6)	1					
weak	12.2 (5.9)		12 (52.2)	1.2	0.4-3.9	0.8			
moderate	11.1 (5.8)		31 (70.5)	2.6	0.9-7.7	0.08			
high	11.3 (5.6)		67 (62.0)	1.8	0.7-4.6	0.2			
**Blood in stool**									
no	11.8 (5.9)	**0.001**	61 (53.5)	1					
yes	11.1 (5.8)		59 (72.0)	2.2	1.2-4.1	**0.01**	1		
**Malaria**							2.3	1.2-4.3	**0.008**
no	11.2 (5.6)	0.8	41 (67.2)	1	0.5-2.0	0.9			
yes	11.2 (5.4)		77 (68.1)	1.0					
**BAZ Score (Wilcoxon)**									
Normal		0.9	82 (59.9)	1					
Too thin			38 564.4)	1.3	0.6-2.3	0.42			
**HAZ Score**									
No	12.0 (6.0)	**<0.001**	36 (47.4)	1			1		
Stunting	11.1 (5.7)		33 (67.4)	2.3	1.7-4.8	**0.03**	1.7	1.1-2.5	**0.007**
Severe stunting	11.2 (5.7)		50 (71.4)	2.8	1.4-5.5	**0.004**			

The third model assessed correlates of stunting (Table 5). Males presented an OR of 2.1 (p=0.01) and weak SCH infection was protective against stunting (OR=0.3; p=0.04)). Children older than 13 years presented 5.3 times more odds to have stunting (Table 5). When multivariate analysis was applied, the factors age (AOR=2.3; p=0.005) and gender (AOR=2.4; p<0.001) were significant.

**Table 5:** Risk factors related to stunting in children attending primary school (3^rd^ to 6^th^ class) in the health zone of Kasansa, DR Congo

Variables	Correlates with stunting				Predictors for stunting		
	n (%)	OR	95% CI	p value	AOR	95% CI	p value
**Gender**							
Male	71 (69.2)	2.1	1.2-3.8	**0.01**	2.3	1.3-4.5	**0.005**
Female	47 (51.6)	1.0					
**Agegr**							
7-9y	8 (34.8)	1.0					
10-12y	55 (57.3)	2.5	1.0-6.5	**0.06**	2.4	1.5-3.9	**<0.001**
>=13y	56 (73.6)	5.3	1.9-14.2	**0.001**			
**SCH infection**							
no	15 (71.4)	1.0					
yes	104 (59.8)	0.6	0.2-1.6	0.3			
**SCH density**							
0	15 (71.4)	1.0					
Weak	9 (39.1)	0.3	0.1-0.9	**0.04**			
Moderate	67 (62.6)	0.7	0.2-2.2	0.54			
High	67 (62.6)	0.7	0.2-1.9	0.4			
**Malaria**							
No	34 (55.7)	1.0					
yes	72 (64.3)	1.4	0.8-2.7	0.3			
**SES**							
<1$	107 (60.8)	0.9	0.3-2.4	0.8			
> = 1$	12 (63.2)	1.0					

The last model assessed correlates of low school performance (Table 6). Only children from farmers had significantly lower school performance (OR: 5.3; p=0.03). When daily expenses were less than 1 USD an OR was found of 2.2 but it was not significant (p=0.1).

**Table 6:** Risk factors related to low school performance in children attending primary school (3^rd^ to 6^th^ class) in the health zone of Kasansa in DR Congo

Variables	*Correlates with low school performance*			
	n (%)	OR	95% CI	p value
**Gender**				
Male	55 (52.8)	0.9	0.5-1.5	0.7
Female	51 (56.1)	1.0		
**Agegr**				
7-9y	11 (47.8)	1.0		
10-12y	49 (51.6)	1.2	0.5-2.8	0.7
>=13y	46 (59.7)	1.6	0.6-4.1	0.3
**SCH infection**				
no	10 (47.6)	1.0		
yes	96 (55.2)	1.4	0.5-3.4	0.51
**SES**				
<1$	99 (56.2)	2.2	0.8-5.9	0.1
> = 1$	7 (36.8)	1.0		
**Profession chief houshold**				
Commercant	1 (50.0)	1.0		
Pastor	14 (50.0)	4.0	0.2-95.8	0.4
Civil servant	2 (20.0)	4.0	0.7-22.3	0.1
Agriculture	87 (56.9)	5.3	1.1-25.7	**0.03**

## Discussion

The present cross-sectional study of children attending primary school (3^rd^ to 6^th^ class) in Kasai Province in DRC showed an alarmingly high prevalence of S. mansoni infection (89.3%) in children aged 6-17 years old. Schistosomiasis has to be acknowledged as a major public health in Kasansa health area. Simultaneously, a high proportion of anaemia (61.4%), stunting (61.0%) was demonstrated and a high proportion of children reported to fail at least one class (54.6%).

The overall prevalence of S. mansoni observed in these children living in Kasansa health area was very high and classified the community of this health area at the high risk zone of schistosomiasis [19]. This high prevalence was being attributable to intense water contact activities of population in the area. Similar results were also observed in Senegal among school children living in the district of Niakhar, region of Fatick [20], an environment similar to Kasansa area where 100% of the population do not have plumbing in their households (table1). As expected, regular contact with river water or the nearest source was the most significant risk factor related to SCH infection. Similar results were found by Alebie *et al*., Amuta *et al.*, and Bowie *et al*. in Ethiopia, Nigeria and Malawi, respectively [21-23]. Children whose parents are farmers might be more at risk, however, this was not observed in this study. Similar findings were reported by Chipeta MG *et al*. (2013), high infection intensity of SCH were compared in farmers and non-farmers groups but farming did not show a significant association with SCH infection [24]. Matthys B *et al*. (2007) as well, showed a similar observation in urban farming communities living in western of Côte d'Ivoire [25]. Since the infection burden is extremely high, nearly all the children were equally at risk to contract a SCH infection. This also suggests that the environment is highly contaminated and several control measures are urgently needed.

The proportion of anaemia in this study was very high (61.4%), the anaemic status could not be correlated to SCH infection, however, Hb concentration was significantly reduced by SCH infected children (p=0.003). Tatala *et al.* reported high prevalence of anaemia among school children along the coast in Tanzania and strong association with schistosomiasis [26]. But concerning the Hb concentration, it was significantly decreased when the intensity of eggs was moderate or high, this is in agreement with Friedman [27]. Self-reported blood in the stool prior to the study also significantly reduced Hb concentration. It is logical consequence that a high burden of infection which will results in a higher proportion of eggs penetrating the wall of the bowel would lead to anaemia [28, 2]. On the other hand, the anaemic status of this study population was only significantly affected by blood in the stool and stunting. Surprisingly, malaria infection was not found to be associated with anaemia in this study; however, stunting or severe stunting was significantly associated with anaemia and reduced Hb concentration. Therefore, it is possible to consider that this anaemia is not associated with malaria but to a chronic process. The high prevalence of schistosomiasis suggests that this illness contributes to these complications [11]. Stunting was also highly prevalent in the study population (61.0%), and boys were more affected than girls (AOR=2.3) (Figure 2). Age was also a significant factor for stunting. As the children grow older they are more at risk to develop stunting (OR from 2.5 to 5.3) which is a logic consequence of a poorly varied diet over a longer period of time. Light egg load was weakly associated to stunting; however, in the multivariate analysis only gender and age were strongly associated to stunting (Table 6). This suggests that a poor varied diet could be the principal cause of stunting in this study population and perhaps not the infections examined here. However, it is important to consider that these risk factors do not act alone. In a community such as Kasansa, it is possible to have a vicious cycle where malnutrition can be the cause of anaemia and vice versa, and this can be aggravated by schistosomiasis.

Finally, factors associated to low school performance were assessed. More than half of the children (54.3%) reported to have repeated a class at least 1 time and older children were more likely to have repeated a class. Farmers' children presented lower school performance with the odds of 5.3 times to have low school performance (p=0.03). Children living in families with low income (<1 USD) have a greater risk for low school performance and indeed in the study populations, 77.8% of the households with daily expenses per person <1 USD were farmers. On the other hand, low income has consequences for the educational attainment of the children because the parents are not always able to pay the tuition fee and the inscription is delayed.

The limitation of the study was the fact that the children were enrolled during a household survey. They were enrolled when they reported to attend 3^rd^ to 6^th^ class of primary school. A selection bias could have occurred compared to selecting the children at school. Therefore, the class distribution was not equal. Older children (15-17 years old) were also found to attend primary school, however, in this environment, it is common that school attendance is not regular, accumulating a delay.

## Conclusion

In conclusion, besides unacceptable prevalence of pathologies like schistosomiasis and malaria, children of Kasansa health area attending primary school also demonstrated a high burden of anaemia, chronic malnutrition and low school performance. Poverty might exacerbate the situation. It is therefore important to perform additional studies to demonstrate the causalities and better distinguish opportunities for control mechanisms for these diseases. Results of this study may be used to direct public health professionals to identify specific intervention strategies against SCH that match the need of the population and therefore may be more effective. One option is to compare the population before and after schistosomiasis mass treatment. Another option is to compare two populations similar in all aspects except that one has schistosomiasis and the other does not; this is difficult to realize. Preventions measures such as snail control, integrated with population drug treatment can contribute to the reduction of schistosomiasis prevalence in Kasansa health area; however, morbidities such as anaemia, malnutrition and low school performance can persist due to the recurring low-level reinfection; so, the treatment may need to continue for a long period to maintain the disease control.

### What is known about this topic

- Schistosomiasis and malaria are a considerable disease burden in schoolchildren in developing countries. They are responsible for significant health and economic burden such as anemia, iron-deficiency, malnutrition and impaired child cognitive development;
- Those two infections show a similar geographic distribution. This geographical overlap between schistosomiasis and malaria, co-infections of these parasites are common which results into various forms of associations, exacerbated health consequences and co-morbidities.

### What this study adds

- Data on co-morbidities associated with schistosomiasis are scarce in the Democratic Republic of the Congo and this study would like to contribute in addressing this gap;
- The very burden and of schistosomiasis was found in this study. Results of this study may be used to direct public health professionals to identify specific intervention strategies against SCH that match the need of the population and therefore may be more effective.

## Competing interests

The authors declare no competing interests.
